# Analysis of Transposon Interruptions Suggests Selection for L1 Elements on the X Chromosome

**DOI:** 10.1371/journal.pgen.1000172

**Published:** 2008-08-29

**Authors:** György Abrusán, Joti Giordano, Peter E. Warburton

**Affiliations:** 1 Department of Genetics and Genomic Sciences, Mount Sinai School of Medicine of New York University, Icahn Medical Institute, New York, New York, United States of America; 2 Laboratory of Aquatic Ecology and Evolution, Department of Biology, University of Leuven, Leuven, Belgium; Stanford University, United States of America

## Abstract

It has been hypothesised that the massive accumulation of L1 transposable elements on the X chromosome is due to their function in X inactivation, and that the accumulation of Alu elements near genes is adaptive. We tested the possible selective advantage of these two transposable element (TE) families with a novel method, interruption analysis. In mammalian genomes, a large number of TEs interrupt other TEs due to the high overall abundance and age of repeats, and these interruptions can be used to test whether TEs are selectively neutral. Interruptions of TEs, which are beneficial for the host, are expected to be deleterious and underrepresented compared with neutral ones. We found that L1 elements in the regions of the X chromosome that contain the majority of the inactivated genes are significantly less frequently interrupted than on the autosomes, while L1s near genes that escape inactivation are interrupted with higher frequency, supporting the hypothesis that L1s on the X chromosome play a role in its inactivation. In addition, we show that TEs are less frequently interrupted in introns than in intergenic regions, probably due to selection against the expansion of introns, but the insertion pattern of Alus is comparable to other repeats.

## Author Summary

Recent experimental findings (for example the ENCODE project) show that many functional non-coding regions of genomes are not conserved across species, making the in-silico discovery of such regions challenging. Transposable elements (TEs), which represent 45 percent of the human genome and typically show no sequence conservation, are particularly intriguing from this point of view, because the highly nonrandom genomic distribution of many TE families in genomes has led to hypotheses that their presence is adaptive and have an epigenetic (regulatory) function. We use a novel approach based on the analysis of interrupted TEs to investigate if repeats are under selection that does not rely on sequence conservation. L1 elements, the most active transposable elements of the human genome, are highly overrepresented on the X-chromosome and were suggested to enhance its inactivation in mammals. We find that the interruption pattern of L1 repeats indicates a function for L1 elements in the inactivation of the mammalian X chromosome. Additionally, we show that a considerable fraction of TEs in introns are under selection for integrity, possibly due to selection on intron size or on TEs themselves.

## Introduction

The activity of transposable elements (TEs) harms their hosts primarily through disrupting coding or other selectively important regions of the genome, and through illegitimate recombination between copies of the repeats. Despite their overall deleterious effect, it is becoming increasingly clear that a considerable fraction of TEs have been domesticated by their hosts, and gained either a regulatory role [1],[2], or less frequently, their sequence has become part of a gene [3],[4]. Functional repeats are typically identified in two ways; either through conservation of their sequence [5],[6], or through co-localization with regions with a known genomic function [7],[8]. However, these methods may not be able to identify many functional repeats in the genome. The initial results of the ENCODE project show that even though up to ∼5% of mammalian genomes might be functional, the sequence of many experimentally determined functional elements is not conserved across species, therefore cannot be identified by sequence conservation [9],[10]. Thus, in the absence of prior information on the functionality of a genomic region, the in-silico discovery of selectively important but not conserved repeats (or genomic regions) remains a challenge.

Analysis of transposon insertions offers at least a partial solution for the detection of such repeats or genomic regions. Recently, Simons et al. [11] identified almost 1000 large, >10 kb regions in mammalian genomes which remained free of transposon insertions in many mammalian genomes, and a considerable fraction of them shows little or no sequence conservation. Since the probability of the random emergence of such high number of transposon-free regions is extremely low, the authors concluded that the maintenance of such regions must involve selection against TE insertions, although their exact function remains unclear. Here we take a further step and use transposon interruptions to analyze the selective constraints on transposable elements themselves. We analyze two TE classes that have been hypothesized to have an epigenetic function, at least in some regions of the genome: L1 repeats in the inactivation of the X chromosome in females of placental mammals [12], and Alus, which accumulate near genes over evolutionary time [13],[14].

**Figure 1:**
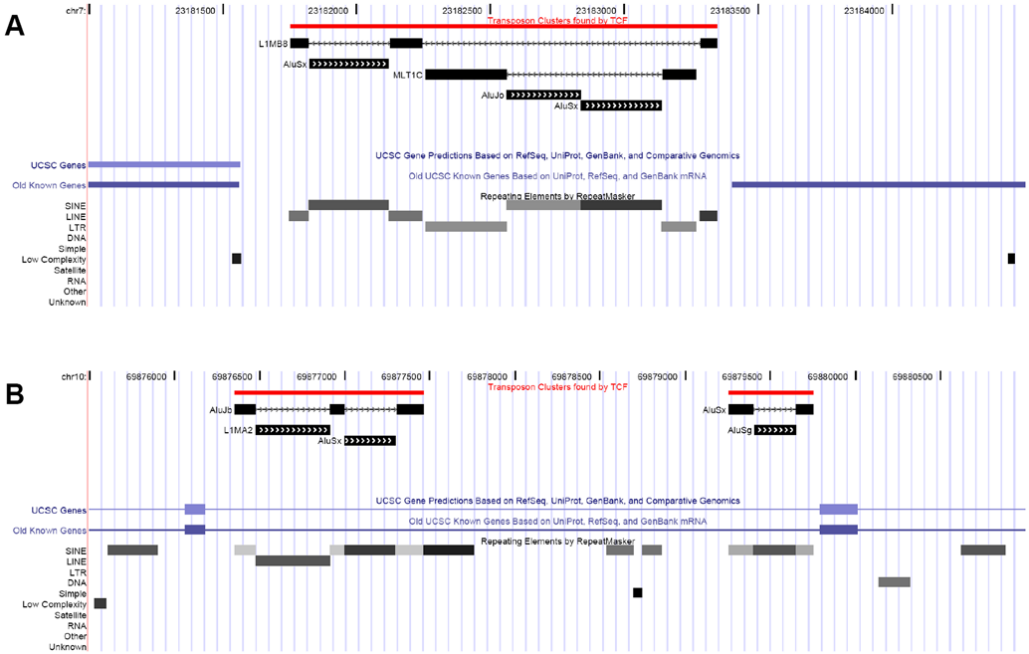
Examples of transposon clusters. A) An ancient L1MB8 element was interrupted by an Alu and a LTR repeat, which in turn was further interrupted by two Alus. B) Alu repeats interrupted by an L1 and other Alus. The UCSC custom track that visualizes TE clusters is downloadable from. http://www.mssm.edu/labs/warbup01/paper/files.html.

TEs on average cover more than 40% of mammalian genomes [15], and remain detectable in primate genomes for up to 200 million years [14]. Since most fixed TE insertions are neutral or nearly neutral, interrupting them by other, younger TEs is also likely to be selectively neutral for the host. In consequence, mammalian genomes contain many nested TE insertions (“TE clusters”), where older TEs are interrupted by younger ones (Figure 1). The analysis of TE clusters can provide information on the evolution of TEs, and has already been used to analyze the relative age of TE families [16]–[20]. Furthermore, analysis of TE interruptions can provide insights on the selective constraints on TEs. Insertion into TE sequences which are beneficial for the host would result in their disruption and loss of function, and individuals carrying such “knock-out” TEs would undergo negative selection and disappear from the population. Thus, analyzing TE interruptions offers a novel way of investigating selective pressures on TEs in mammalian genomes. In addition to the identification of functional but non-conserved regions, in many genomes inference about conservation is complicated by the high spatial heterogeneity of substitution rates in different parts of the genome [21],[22]. Factors that lead to such heterogeneous substitution rates are the large variability of chromosome size, for example in avian and reptile genomes where the size of chromosomes spans two orders of magnitude [23],[24], or the complex evolutionary history found on the sex chromosomes[25],[26]. In such organisms and genomic regions the analysis of TE insertions may be a valuable tool in the detection of functional repeats, supplementing standard methods based on sequence comparison.

**Figure 2:**
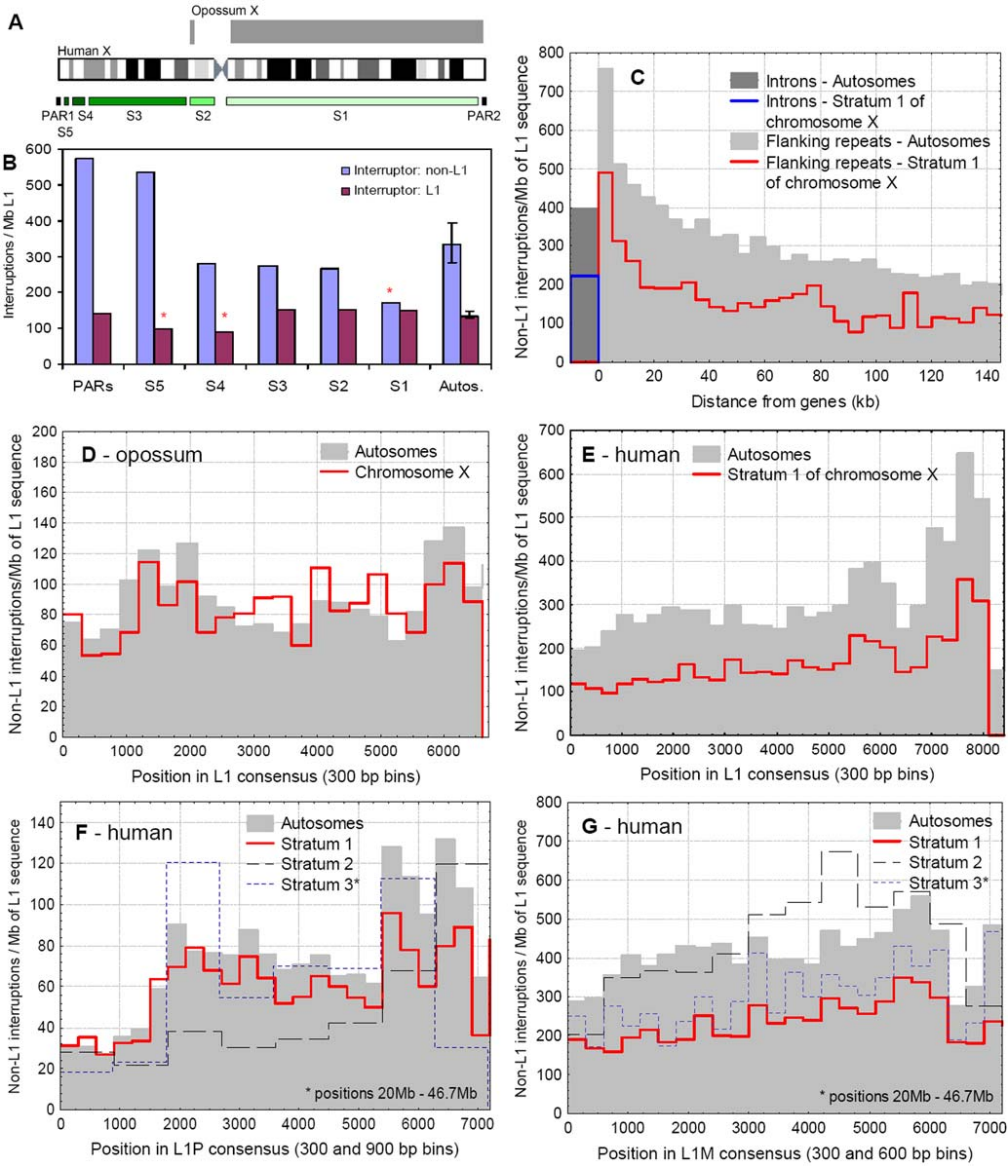
The pattern of interruptions of L1s on the autosomes and X chromosomes. A) The approximate position of evolutionary strata and the pseudoautosomal regions on the human X chromosome and the homology with the X chromosome of the opossum. The largest and oldest stratum on the human X corresponds to the opossum X. B) The frequency of interruptions (per megabase of L1 sequence) of L1 elements in the different evolutionary strata of the X chromosome, and the median of autosomes (error bars indicate quartiles). Clusters containing at least one L1, and only non-L1 interruptions were analysed separately, as the interruption of an L1 repeat by another L1 may not result in loss of function of the locus. On the oldest evolutionary stratum (S1) L1s are significantly less frequently interrupted than on autosomes. C) The frequency of non-L1 interruptions in introns and intergenic sequences of the autosomes and stratum 1 of the X chromosome. The frequency of interruptions in intergenic sequences is grouped into 5 kb bins, as a function of the distance from the nearest gene. D) The frequency of L1 interruptions by non-L1 repeats on the opossum autosomes and X chromosome. The positions of interruptions are grouped in 300 bp bins across the consensus sequence. There is no significant difference in the frequency of interruptions between the autosomal and X-linked L1s. E) The frequency of interruptions by non-L1 repeats of all human L1 elements on the autosomes and stratum 1 of the X chromosome. There are two clear patterns: L1s on the autosomes are more frequently interrupted than on the S1 of the X chromosome, and the 3′UTRs of the L1 consensus sequence are targeted more frequently than other regions. However, no particular region of the L1 consensus is protected from interruptions. F) The frequency of interruptions by non-L1 repeats into the primate specific L1P clade on the autosomes and the three oldest strata (S1–S3) of the X chromosome. Interruptions are grouped into 300 bp bins on the autosomes and S1, and into 900 bp bins on the S2 and S3, due to their smaller size. Despite the accumulation of L1Ps on the X (which has been interpreted as a signature of their function in X inactivation), with the exception of the S2 region, there is only a minor difference between the frequency of interruptions on the autosomes and the X. Although there is a large difference between the frequency of interruptions in the 5′UTR, ORF and 3′UTR of the L1 consensus sequence, no region of L1Ps is free from interruptions. G) The frequency of interruptions by non-L1 repeats into the mammalian-wide L1M clade of L1s on the autosomes and the three oldest strata (S1–S3) of the X chromosome. Interruptions are grouped into 300 bp bins on the autosomes and S1, and into 900 bp bins on the S2 and S3, due to the smaller number of repeats. Unlike the primate specific L1Ps, L1Ms on the X chromosome are much less interrupted than on the autosomes.

In mammals, due to the XY sex determination system, one of the X chromosomes in females is inactivated during early embryogenesis [27],[28]. Inactivation evolved separately in marsupials and placental mammals [29]; in marsupials strictly the paternal X chromosome is inactivated [30], while in placental mammals the inactivated X chromosome is selected at random [27],[28]. In humans X chromosome inactivation is mediated by a 17 kb long non-coding RNA produced by the Xist gene [31], which appeared prior to the mammalian radiation [29],[32], but is absent in marsupials [30]. Inactivated genes are not evenly distributed on the human X chromosome, but instead are mostly located on the oldest evolutionary “strata” (S1–S3) of the chromosome [33] (see also Figure 2), which largely correspond to the opossum X chromosome [30]. The exact mechanism of inactivation is not known, but the higher than average abundance of L1s on the X chromosome[12],[34], particularly near inactivated genes have led to the hypothesis that L1s have a role in the inactivation process, by serving as “way stations” for the spread of the inactivation signal. Recent computational analyses show that the inactivation status of X-linked genes can be predicted by the neighboring repeats [35],[36]. However, the sequence conservation of L1s on the X chromosome does not differ qualitatively from the autosomes, and it is also unclear whether the unique patterns of repetitive element distributions on the X chromosome are the cause, or consequence of inactivation (or both).

With more than one million copies, Alus are the most abundant TEs in our genome [14],[37]. They are primate specific, parasitize active L1s for replication [38], and insert primarily into gene-poor, AT-rich regions of the genome. However, the genomic distribution of Alus changes with their age; in contrast to the youngest insertions the vast majority of Alu repeats are present in GC and gene-rich regions of the genome [13],[14]. The high density of Alus near and within genes has led to the hypothesis that many of these insertions might be preferentially retained in the genome due to a not yet fully identified function [14],[39],[40].

In this paper we test whether L1s on the X chromosome and Alus near genes are less interrupted than expected by their genomic abundance. We interpret reduced amounts of interrupted TEs as a signature of selection for the integrity of the TE sequence in that region (selection against “knock-out” TEs).

## Results

### L1s on the X Chromosome

We investigated the selective constraints on L1 elements on mammalian X chromosomes by examination of the frequency of interrupted L1s. We analysed the evolutionary strata of the X chromosome independently, to account for their different evolutionary histories and proportion of inactivated genes. The clusters of interrupted L1s were categorised into two groups, depending on whether L1s were interrupted by L1s, or by different types of repeats (Figure 2B). (We made this distinction because interrupting a TE by a similar TE may not result in loss of functionality of the locus). The frequency of non-L1 interruptions changes across the human X chromosome, it is highest on the still recombining pseudoautosomal regions and the youngest evolutionary strata, and lowest on the oldest stratum, where the frequency of interrupted L1s is significantly lower than on the autosomes (p = 0.0012, Wald-Wolfowitz runs test [WWrt], Figure 2B). In contrast, there is no such trend in the clusters containing L1s interrupted by other L1s; the frequency of these interruptions in the oldest strata, and in the pseudoautosomal regions is comparable to the genomic median (p<0.05 only for S4–S5, WWrt, Figure 2B).

In the opossum genome we found no significant differences in the frequency of interrupted L1 elements between the autosomes and the X chromosome (p = 0.23, Wilcoxon signed rank test [Wsrt], Figure 2D). In contrast, on Stratum 1 of the human X, L1s are approximately twofold less frequently interrupted compared with the autosomes (p<0.001, Wsrt, Figure 2E). The frequency of L1 interruptions declines with the distance of the repeats from the genes, both on autosomes and the X chromosome (p<0.001, Wsrt, Figure 2C).

There are large differences between the different L1 families: the relatively young, primate specific L1P families are interrupted on the S1 and S2 strata of the X chromosome at significantly lower rates than on the autosomes (Figure 2F, p<0.001, p = 0.017 and p = 0.26 for S1, S2 and S3 respectively, Wsrt, see also Figure 4B). However, the difference is small in comparison with the older L1M families, which were active mainly before the mammalian radiation, and are much less interrupted on the human X than on the autosomes (Figure 2G, p<0.001, p = 0.55 and p<0.001 (Wsrt) for S1, S2 and S3 respectively. (Note that the gene density of S2 is higher than the genomic average). The 5′UTRs, ORFs and 3′UTRs of L1s are interrupted by non-L1 repeats at different frequencies, particularly in the case of primate specific L1s (Figure 2F and G; the frequency of interruptions is calculated per total amount (base pairs) of the L1's in each window, and do not simply reflect the different abundance of these regions). The pattern of interruption is qualitatively similar on the autosomes and X chromosome, and no specific regions within L1s are free of interruptions compared with the autosomes.

**Figure 3:**
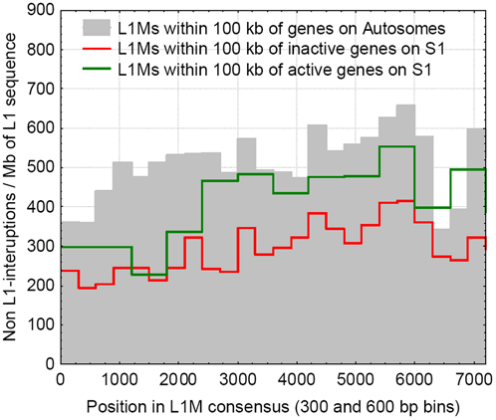
The frequencies of interruptions into L1Ms, within 100 kb of genes, for the genes that escape inactivation on the S1, are inactivated on the S1, and on the autosomes. L1Ms near genes escaping inactivation are interrupted at a higher frequency than near the inactivated ones, but at a lower rate than on the autosomes.

The inactivation of genes on the X is incomplete, and several genes escape inactivation (at least partly), even on the oldest stratum (S1) of the X chromosome. We compared the frequency of interruptions in S1 that are found within 100 kb of both inactivated genes and genes that escape inactivation. We find that the L1M repeat sequence in the vicinity (and within) genes escaping inactivation is interrupted at significantly higher rates than L1Ms near genes that are subject to inactivation (p = 0.0019, Wsrt, Figure 3), but at a somewhat lower rate than on the autosomes (p<0.001, Wsrt, Figure 3), further corroborating the relationship between the presence of uninterrupted L1s and inactivation.

### Frequency of Interruptions vs. Their Distance from Genes

**Figure 4:**
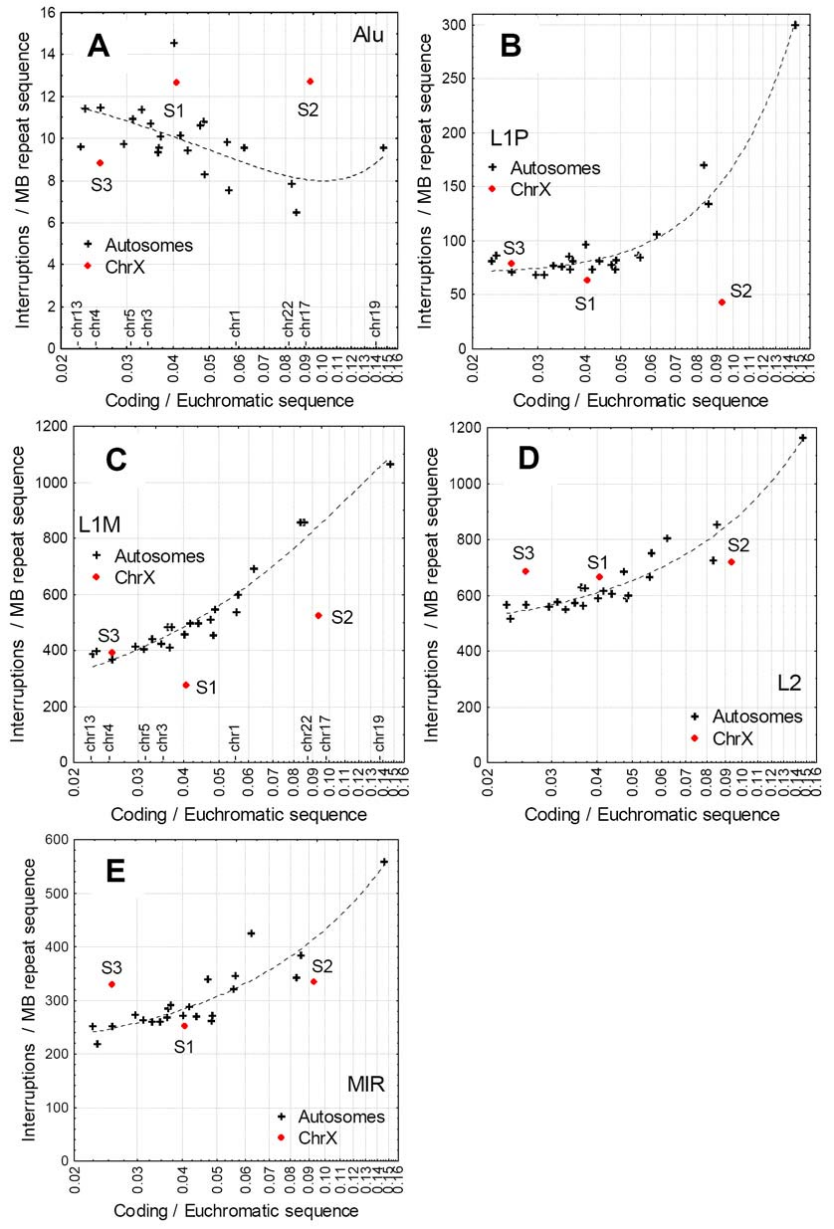
Correlations between the density of coding sequence in the euchromatic region of chromosomes (coding sequence/non-repetitive sequence), and the frequency of interruptions of the main non-LTR repeats. The positions of selected chromosomes are indicated on the x-axis on panels A and C. Note the logarithmic X axis. Each cross represents an autosome, the three strata (S1–S3) of the X chromosome that contain the most inactivated genes are shown in red. With the exception of Alus, repeats are interrupted more frequently on gene dense chromosomes, and unlike L1s, other non-LTR repeats do not show a depletion of interruptions in the S1–S3 strata.

Since the frequency of interrupted repeats shows clear dependence on the distance of the interrupted repeat from genes (Figure 2C, Figure 4B, C), the reduced frequency of L1 interruptions on the X chromosome could be a simple by-product of a lower than average gene density on the oldest evolutionary strata. In addition, if L1s are the only or main repeat type involved in X inactivation, than only L1s should show reduced frequency of interruptions on the X chromosome but not other non-LTR repeats. We tested these hypotheses by analyzing the frequency of the interruptions of the most abundant non-LTR repeat classes of the human genome (Figure 4) on each human chromosome, using the percentage of coding sequence in the euchromatic sequence as a covariate. With the exception of Alus, the frequency of interruptions of each type of TE correlates positively with the density of coding sequence on the chromosomes. This can be explained by the lower average distance of the repeats to coding regions. In the case of L1s the S1 and S2 regions of the X chromosome are clear outliers, indicating that the lower frequency of interruptions cannot be explained with low density of genes on these strata (Figure 4), while L2s and MIRs do not show this effect.

### Alu Repeats and the Frequency of Interruptions in Introns

**Figure 5:**
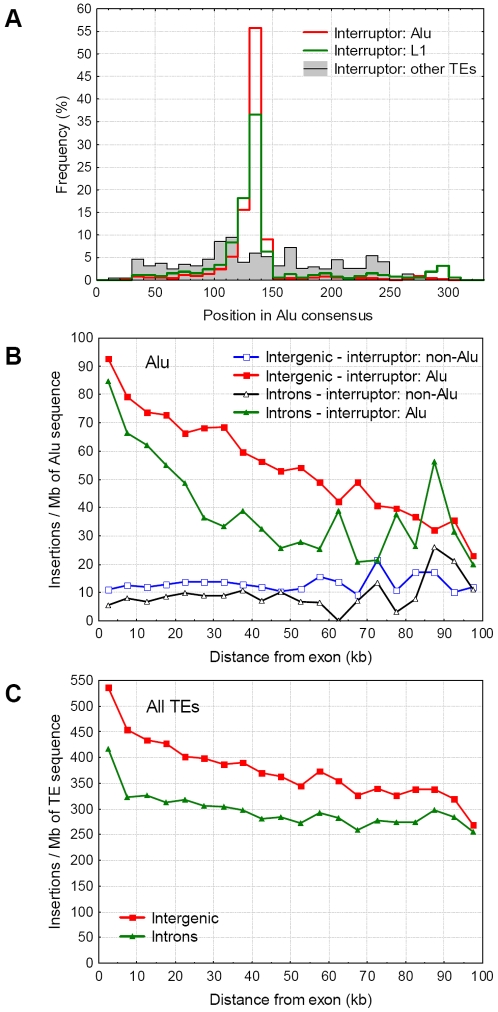
Interruption pattern of Alu repeats on autosomes. A) The distribution of interruptions within the Alu consensus sequence. The vast majority of Alus and L1s insert into the poly-A stretch of the linker region, while the insertions of other repeats are distributed equally across the repeats. B) The distribution of Alu interruptions (standardized with the amount of Alu sequence) in introns and intergenic regions as a function of the distance from the nearest exon or gene, in 5 kb bins. The distribution of Alus interrupted by Alus and other repeats are indicated separately, both for intergenic and intronic repeats. C) The distribution of all TE interruptions in introns and intergenic regions.

A relatively large number (>21 000) of Alus are interrupted in the genome, and the vast majority of the interrupters are other Alus. Due to the target specificity of the L1 integrase which Alus use (TT|AAAAA), most Alus are interrupted in the polyA stretch of the linker region between the two Alu halves (Figure 5A). In contrast, interruptions of Alus by TEs other than Alus or L1s are spread out approximately evenly across the Alu consensus sequence (Figure 5A). The frequency of interruptions of Alu's by other Alus increases nearer to genes and exons, while the frequency of Alus interrupted by non-Alus remain relatively constant (Figure 5B). We find a clear difference between intergenic and intronic Alus; in introns Alus are interrupted with a considerably lower frequency both by Alus (p<0.001, Wilcoxon matched pairs test) and other repeats (p = 0.009, Figure 5B), suggesting that interrupting a fraction of Alus in introns is deleterious. However, this pattern is not restricted to Alus, a qualitatively similar trend is present for other repeats (Figure S1) and the combined dataset of all TEs (p<0.001, Figure 5C), indicating an overall selection against disrupted repeats in introns, that includes but is not specific for Alus.

## Discussion

### L1s on the X Chromosome

Recombination between the human X and Y chromosomes ceased gradually in the last ∼300 my, leading to two pseudoautosomal regions and five evolutionary strata (S1–S5) on the X (Figure 2A); the largest and oldest of them (S1) roughly corresponds to the opossum X chromosome (Figure 2). The distribution of inactivated genes is not random between the strata; on the oldest one (S1) the vast majority of the genes are inactivated, while on the youngest strata (S4–S5) most of the genes escape inactivation [33].

Our results support the hypothesis that L1 repeats have a role in the spread of the inactivation signal on the X chromosome. The low frequency of L1 interruptions in strata with high number of inactivated genes suggests that there is selection against L1 interruptions in these regions, and individuals with interrupted L1s near inactivated genes were removed from the population more efficiently than individuals carrying interrupted L1s on the autosomes, where such interruptions are likely to be neutral.

Theoretically the lower frequency of interruptions could emerge also as the result of the relaxation of selection on the X chromosome, both due to its reduced rate of recombination or inactivation. A recent study have demonstrated that full length L1s are subject to negative selection in the human genome while truncated L1s are essentially neutral [41]. Weaker selection would lead to less efficient removal of “standalone”, in particular full length repeats from the chromosome, and consequently result in a lower frequency of interrupted repeats. However, a process like this would influence all types of repeats not just L1s, and other, equally old non-LTR repeats like L2s and MIRs are not less interrupted on the X chromosome than elsewhere in the genome (Figure 4). This is probably true for other chromosome-wide processes that are not specific for L1 elements, such as paternal transmission of Alu repeats [4],[42]. Additionally, ancient L1Ms which have essentially no full length copies and on average have lower insertion length than L1Ps show the strongest pattern.

The timing of the evolution of random, Xist-mediated inactivation [29],[43] is consistent with our results. In the opossum genome, where there is no random X inactivation and the Xist gene is absent [30], L1s show equally frequent interruptions on the X chromosome and on the autosomes (Figure 2D). However, in humans the L1M families, which were active before the mammalian radiation and present when Xist-mediated inactivation evolved, are less frequently interrupted on the X than on the autosomes (Figure 2). The difference in the frequency of interruptions between the S1 and the autosomes is much smaller for the primate specific L1P families, despite the strong accumulation of L1Ps on the X chromosome. Since most arguments about the putative L1 function in inactivation were based on the increased L1P abundance on the X chromosome [12],[34],[44], the small difference in the frequency of L1P interruptions between the autosomes and the S1 is surprising. One possibility is that the accumulation of L1s on the X is a consequence and not the cause of the inactivation, due to a so far unknown mechanism. On the other hand, the primate specific L1P families are relatively young and are interrupted at much lower frequency than the mammalian wide L1M families (Figure 2), and most L1P insertions are also considerably longer than L1Ms (∼1400 bp vs. ∼700 bp respectively). Due to the low frequency of interruptions (approximately 10% of L1P repeats are interrupted, while almost 40% of L1Ms) the amount of uninterrupted sequence may still be high enough to influence the spread of the inactivation signal; thus, despite the fact that on the S1 region we found no clear support for it, the conclusion that primate specific L1s have no influence on X inactivation is premature.

### Gene Density, Distance from Genes and the Frequency of TE Interruptions

The frequency of interrupted repeats within a TE family is largely determined by the age of the family; older families had more time to accumulate insertions and are more interrupted than young ones; this has already been used by our group to determine the age of mammalian repeats [20]. However, besides age, gene density has also significant influence on the frequency of interrupted repeats. We found that in the vicinity of genes TEs are likely to be more interrupted than in gene poor regions (Figure 2), and in consequence gene dense chromosomes have higher frequency of TE clusters (Figure 4). We propose two mechanisms which can cause this pattern. In gene dense regions the likelihood that a new insertion into the euchromatic sequence will be deleterious due to the disruption of a coding or regulatory sequence is high, while inserting into another TE is likely to be neutral (with the exception of specific regions where TEs acquired some function, like exapted repeats or the X chromosome). This is likely to result in increased frequency of interrupted repeats close to genes. A second mechanism that can result in the depletion of TE clusters in gene-poor regions is illegitimate recombination between repeats [45]–[49], particularly Alus. Illegitimate recombination is probably the key process behind the large spatial variability of TEs in the genome, and particularly the distribution of Alus is modified by its effects [45],[46],[48],[50]. Due to their large numbers Alus are the most frequent interrupters in the genome, and if the probability of ectopic recombination between Alu elements is larger than between the original, uninterrupted repeats, than illegitimate recombination between Alus reduces also the amount of TE clusters, because it results in deletions [51],[52] which can contain a part of the cluster. Since the likelihood that deletions are neutral and will reach fixation is highest in gene poor regions, this process leads to a positive correlation between gene density and frequency of TE interruptions, similarly to the density of Alu repeats.

### Alu Repeats and the Frequency of TE Interruptions in Introns

The vast majority of Alus are interrupted in their A-rich linker region that connects the two GC rich arms or the repeat (Figure 5A). This pattern can be easily explained by the insertion preference of the repeats; both Alus and L1s target TT|AAAAA sites, while interruptions of all other repeats with different target site specificity are not clustered at the linker region. The lower frequency of interrupted Alus and other TEs in introns than in intergenic regions suggests that intronic TE interruptions may be deleterious. This is in agreement with the findings that several Alu containing exons are alternatively spliced, and suggestions thus Alus may have a profound influence on the human transcriptome [53],[54]. In addition, a recent study by Gal-Mark et al. [55] have demonstrated that both arms of Alus are used in this process, and experimentally increasing the distance between them results in deleterious splicing. Since the majority of Alus are interrupted exactly in the linker region between the two Alu arms (Figure 5A), these findings provide an elegant example of the loss of biological function due to interruption of a repeat. However, only 0.2% of Alus appear to be exonised [54], and the pattern we observe is not specific for Alus (Figure 5C, Figure S1), thus this mechanism alone is not sufficient to explain the low frequency of interruptions in introns, or the accumulation of Alus in gene-dense regions.

TEs show biases in their orientation in introns due to selection against Alus, L1s and in particular LTR insertions in the forward direction, because these repeats can interfere with transcription [56]. In consequence, in introns the neutrality of an insertion depends on the orientation of the TE as well, and in consequence a fraction of TE insertions in the forward direction is likely to be deleterious, even if they interrupt other repeats. This process results in a lower frequency of interruptions in introns, however, it can account for less than a half of the difference between the frequency of interruptions in introns and intergenic regions (Figure S2), because the bias in interruptions is much stronger than in the number of TEs.

We see at least two additional processes that may lead to reduced frequency of interrupted repeats in introns. First, introns may be selected for small size [57],[58], and since new TEs increase intron size they are weakly deleterious, even if they disrupt a transposable element. Thus, unlike in intergenic regions an insertion into a transposable element isn't neutral in introns, leading to a higher probability that such nested insertions will be lost during evolution. This hypothesis does not assume that Alus or other repeats have any specific benefit for the host, and it predicts that the effect will be the strongest in short introns of highly expressed genes, where selection for small intron size is the strongest [58]. This prediction is consistent with our findings: the difference in the frequency of interruptions between intronic and intergenic repeats is the largest in the vicinity of exons, and gradually declines with the increasing distance from exons (Figure 5C).

An alternative hypothesis is that selection acts on some of the TEs themselves, and a fraction of the TEs within introns are beneficial for the host, most likely due to their effect on gene expression. This is consistent with recent findings, which indicate that a large number of TEs are involved in gene regulation through cis natural antisense transcripts, and that 98.2% of such repeats are present in introns [59].

An interesting pattern in the data is the much more pronounced accumulation on self-interrupted Alus near genes than Alu sequence (Figure 5). The large scale shift in the frequency of self-interrupted repeats spanning more than 100 kb supports the studies showing that the Alu distribution in the genome is significantly shaped by illegitimate recombination [45],[46],[48]. Illegitimate recombination between nested Alus accounts for 8% of Alu-Alu recombination mediated deletions in the chimpanzee [50], but only 1.8% of Alus contain a self-insertion in the human genome, thus deletions caused by self-interrupted Alus are 4.3 times more frequent than between individual Alus. This is expected to reduce the density nested Alus, particularly in gene poor regions, where such deletions are less deleterious and can reach fixation. The reason for the higher frequency of recombination between nested alus is probably their length because ectopic recombination depends on the length of a repeat [60], and tandemly repeated sequences are particularly prone for it (a nested Alu insertion contains 4 almost identical arms). Taken together, our data confirm that the variability in the abundance of Alu repeats in primate genomes is caused by the frequency of (illegitimate) recombination.

### Conclusions

1. In regions of the X chromosome which are subject to inactivation (strata 1 to 3), L1 elements, primarily L1Ms are interrupted at lower frequency than on autosomes or other, more active regions of the X chromosome. Assuming that lower than expected frequency of interruptions indicate selection, our analysis suggests that the ancient L1M repeats are utilized by the inactivation mechanism, while we found support for such function for the primate specific L1Ps only on the S2. This is consistent with the phylogenetic distribution of X inactivation, which probably evolved before the mammalian radiation (and the appearance of the L1P clade).
2. On the X chromosome of the opossum which has no random inactivation, lacks the Xist, and is largely homologous to the oldest evolutionary stratum of the Human X, the pattern of L1 interruptions is similar to the autosomes.
3. The frequency of interrupted TEs is highest near genes and exons, probably due to the higher likelihood of deleterious insertions in gene dense regions, and the more frequent loss of TE clusters from gene poor regions via non-homologous recombination between repeats.
4. TEs are less interrupted in introns than in intergenic regions, possibly due to selection on intron size.
5. The analysis of TE interruptions appears to be a useful method to gain insights on the selective constrains on genomes. The method is clearly not as informative as inference from sequence conservation; its main limitations are that it cannot provide information on individual TE copies, can be used only in repeat rich genomes, and identifying the real target of selection (e.g. intron size vs. repetitive elements) may need additional work. However, its major advantage is that it does not rely on any assumption on substitution rates, prior knowledge on functionality, or on the assumption that functional copies of TEs are conserved, which make it a valuable tool for analyses where these assumptions are uncertain.

## Materials and Methods

### Data Sources

The following files were downloaded from the UCSC Genome Browser: the RepeatMasker annotation files for the human (hg18) and opossum (monDom4) genomes, which provide the coordinates of repetitive elements, and the UCSC known-gene file for the human genome that provides the genomic coordinates of genes. The coordinates of the evolutionary strata of the X chromosomes were taken from Carrel and Willard [26]. In the first step of the analysis we integrated these datasets, and determined the position (intergenic, intronic), distance (bp), and orientation (the same or opposite strand) of each transposable element in relation to its neighboring genes. The inactivation status of genes on the X chromosome is available from Carrel and Willard [33]; we used their coordinates in the hg18 draft (UCSC) of the human genome, and excluded genes that were not present in the databases of UCSC. Genes that showed activity in at least 30% of the cases were considered as escaping inactivation. The amount of coding sequence for each chromosome (Figure 5) was determined using the knownGenes dataset of UCSC, while the amount of euchromatic sequence was determined from the raw sequence files.

A large fraction of transposable element insertions are fragmented: most old repeats which have originally inserted into the genome have been split into several fragments, either due to recombination and short insertions or due to insertions of other, younger transposable elements into their sequence. Defragmentation is the reconstruction of the original insertion from its fragments; we defragmented TEs using Transposon Cluster Finder (TCF), a program recently developed by our group [20], and identified interrupted transposons – TEs that inserted into other, older TEs. TCF supports two methods of identifying interruptions; one by its native algorithm (described in detail in [20]), and it can also use the defragementation information (IDs) present in the RepeatMasker output. We used the native defragmentation algorithm of TCF in our analyses (the number of TE clusters found by these two methods are comparable, and they lead to similar conclusions), and determined the key characteristics of transposon clusters: the positions of interruptions in the consensus TE sequence, and the interrupting repeats.

### Data Analysis

Since the probability of being interrupted depends on the length and density of the repeats (the likelihood that an individual TE insertion will be interrupted is higher for longer repeats), and both vary between chromosomes, (for example, L1 insertions are longer on the X chromosomes than on the autosomes [61]), we standardized the frequency of interruptions with the length of the repeats within the analyzed regions. The frequency of interruptions was calculated as the number of interruptions within the copies of a TE family, divided by the summed length of insertions of the same TE family in the analyzed region.

We also determined the frequency of interruptions across the repeat consensus sequence, and its dependence on the distance from exons. This was calculated as follows: for Figures 2C and 5B–C the amount of TE sequence falling into 5 Kb bins counted from gene and exon boundaries were calculated. TEs falling into more than one bin were split and only the fraction of the repeat overlapping with the bin was added to the amount of TE sequence in that bin. For Figures 2D–G and 3, the sequence of L1s was split into 300, or on the smaller evolutionary strata of the X chromosome to 600–900 bp long bins along the consensus sequence, to examine the frequency of interruptions in different regions of repeats. The insertion profile of Alus across the consensus sequence (Figure 5A) was not standardized with Alu length, because Alus do not show biases in their sequence distribution comparable to L1s. The frequency of TE interruptions was determined separately for the clusters that did and did not contain a “self-insertion” (i.e. an L1 repeat interrupted by a younger L1).

The abundances of repetitive elements show large scale correlations in the genome [13],[14]; for example Alus are most abundant near genes while L1s in gene poor areas, due to differences in the rate of repeat removal by ectopic recombination and small deletions [46],[62] in different genomic regions. The rate of repeat loss is in turn determined by recombination rate and density of coding sequence, and has a large effect also on the frequency of interrupted repeats. To account for the combined effects of gene density, deletions (TE-loss), and distance to genes we included a covariate to the analysis, the ratio of coding and euchromatic sequence, which explains a large percentage of the variance (Figure 4).

The sizes of the oldest evolutionary strata (S1–S2–S3) are very different; the S1 and S3 are comparable to the small autosomes, but the S2 is only 15.7 Mb long. Since the variability of the frequency of interruptions is expected to be higher for smaller genomic regions, we divided the genome into 207 15.7 MB non-overlapping windows and calculated the frequencies of interrupted L1s and the density of coding region in them. The regressions between the frequency of interruptions and fraction of coding sequence (Figure S3) show higher variance than the plots containing data from the autosomes, nevertheless the pattern is qualitatively similar, and the S2 is significantly less interrupted than regions of comparable length on the autosomes (t-tests, p<0.001 both for L1P and L1M, using second order polynomials to estimate regression residuals).

## Supporting Information

Figure S1 A) The distribution of L1 interruptions in introns and intergenic regions. The distribution of L1s interrupted by L1s and other repeats are indicated separately, both for intergenic and intronic repeats. B) The distribution of L2 interruptions in introns and intergenic regions. C) The distribution of MIR interruptions in introns and intergenic regions. Due to the very low number of self-interruptions of MIRS and L2s (e.g. a MIR interrupted by another MIR) these were not plotted.
(0.33 MB TIF)

Figure S2 Biases in the frequency of TE insertions and abundance. The expected abundance of TEs in introns is two times the number of insertions in the opposite direction to the embedding gene, because many repeats in the forward direction interfere with transcription and are selected against. In consequence, this bias results in a lower frequency of interrupted repeats in introns than in intergenic regions were there is no such interference. However, the bias in the frequencies of interruptions is much stronger than in repeat abundances, suggesting that other processes significantly influence the frequency of interruptions in introns.
(0.18 MB TIF)

Figure S3 Regressions between the frequency of interruptions and fraction of coding sequence for 15.7 MB regions in the genome and the S2.
(0.21 MB TIF)
