# Supplementary figures and images for: Analysis of Transposon Interruptions Suggests Selection for L1 Elements on the X Chromosome
Source: PLoS Genet. 2008 Aug 29;4(8):e1000172. doi: 10.1371/journal.pgen.1000172 (PMC2517846; doi:10.1371/journal.pgen.1000172)

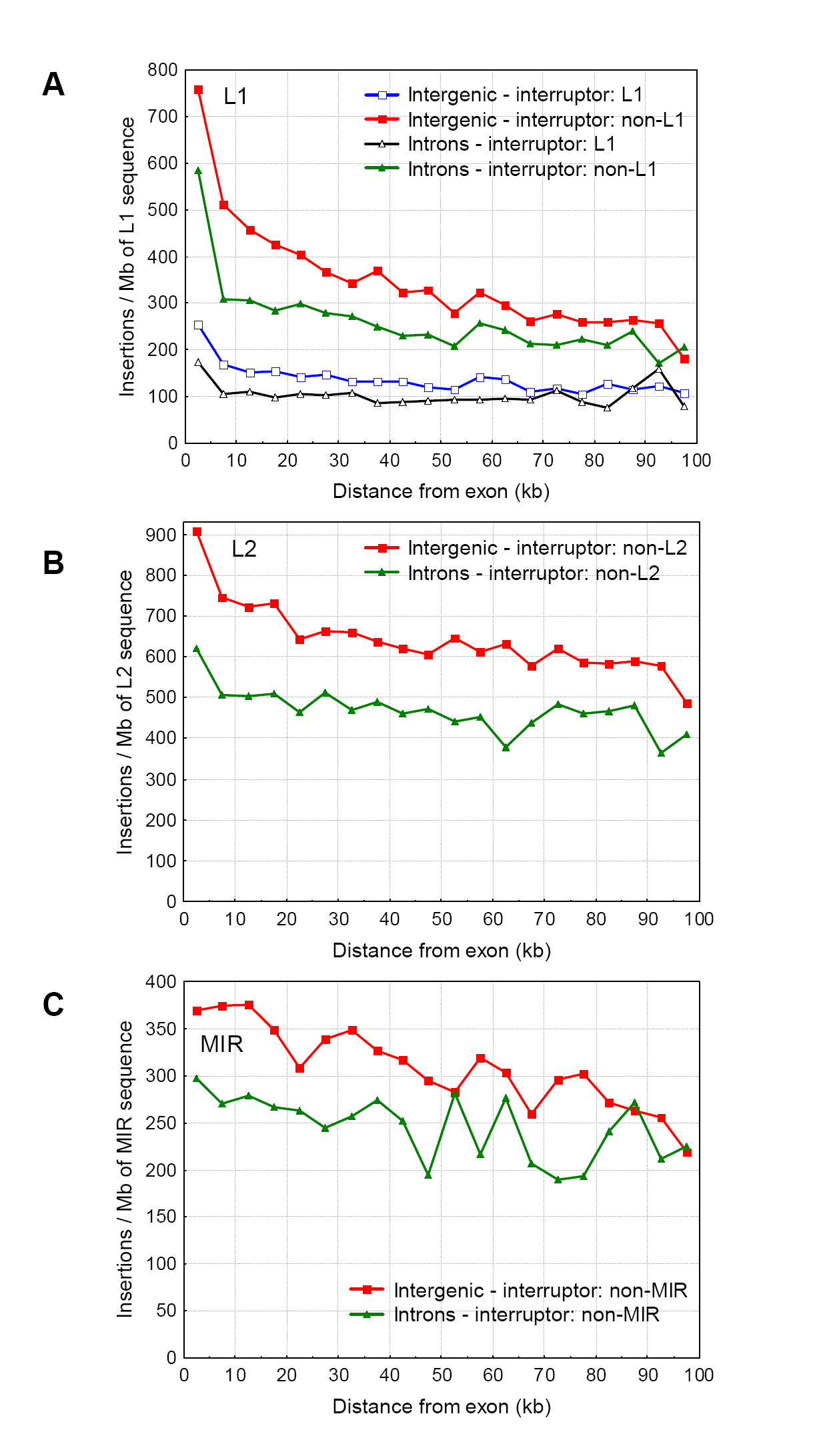

Supplement: Figure S1 — A) The distribution of L1 interruptions in introns and intergenic regions. The distribution of L1s interrupted by L1s and other repeats are indicated separately, both for intergenic and intronic repeats. B) The distribution of L2 interruptions in introns and intergenic regions. C) The distribution of MIR interruptions in introns and intergenic regions. Due to the very low number of self-interruptions of MIRS and L2s (e.g. a MIR interrupted by another MIR) these were not plotted. (0.33 MB TIF) [file pgen.1000172.s001.tif]

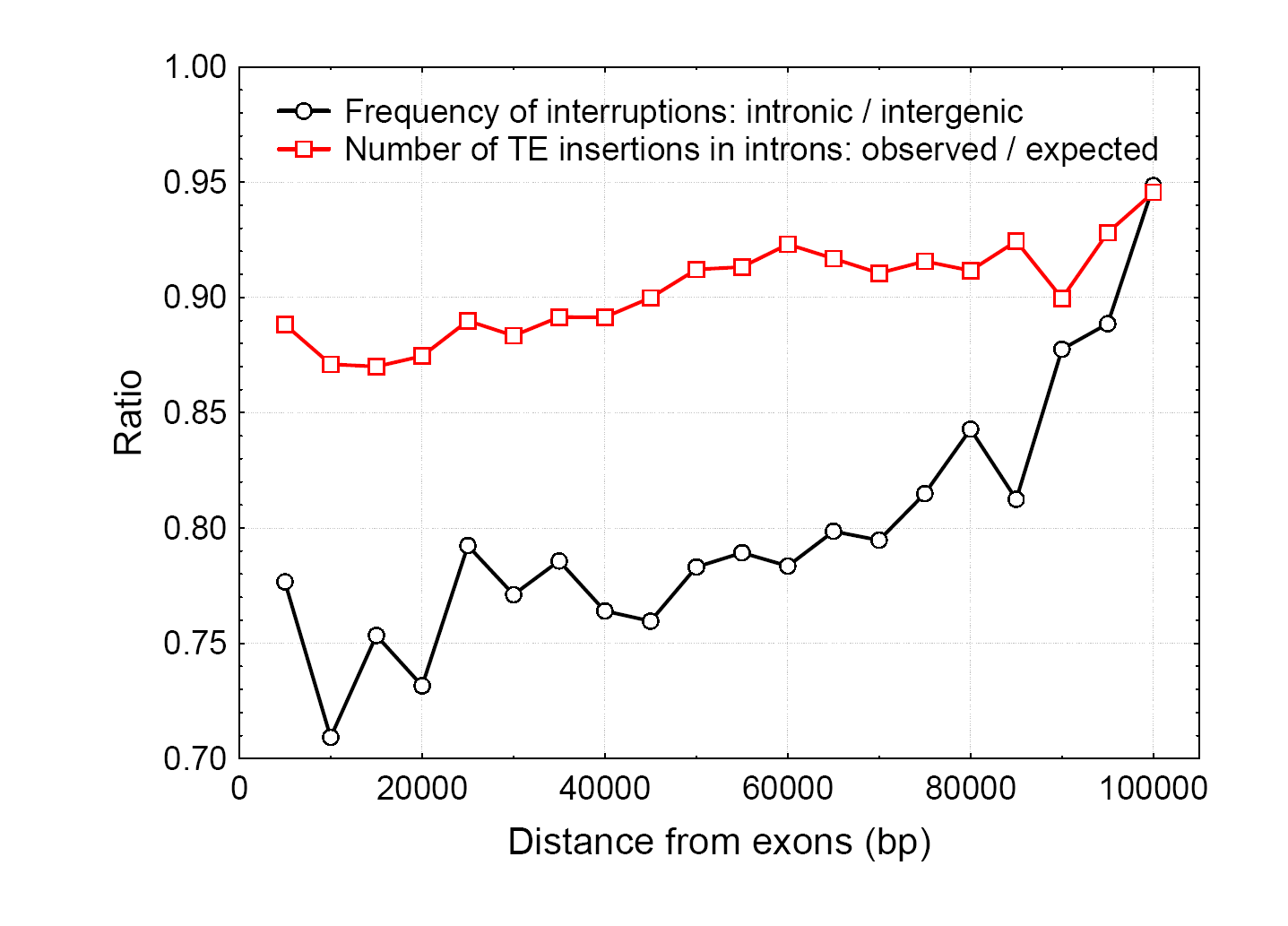

Supplement: Figure S2 — Biases in the frequency of TE insertions and abundance. The expected abundance of TEs in introns is two times the number of insertions in the opposite direction to the embedding gene, because many repeats in the forward direction interfere with transcription and are selected against. In consequence, this bias results in a lower frequency of interrupted repeats in introns than in intergenic regions were there is no such interference. However, the bias in the frequencies of interruptions is much stronger than in repeat abundances, suggesting that other processes significantly influence the frequency of interruptions in introns. (0.18 MB TIF) [file pgen.1000172.s002.tif]

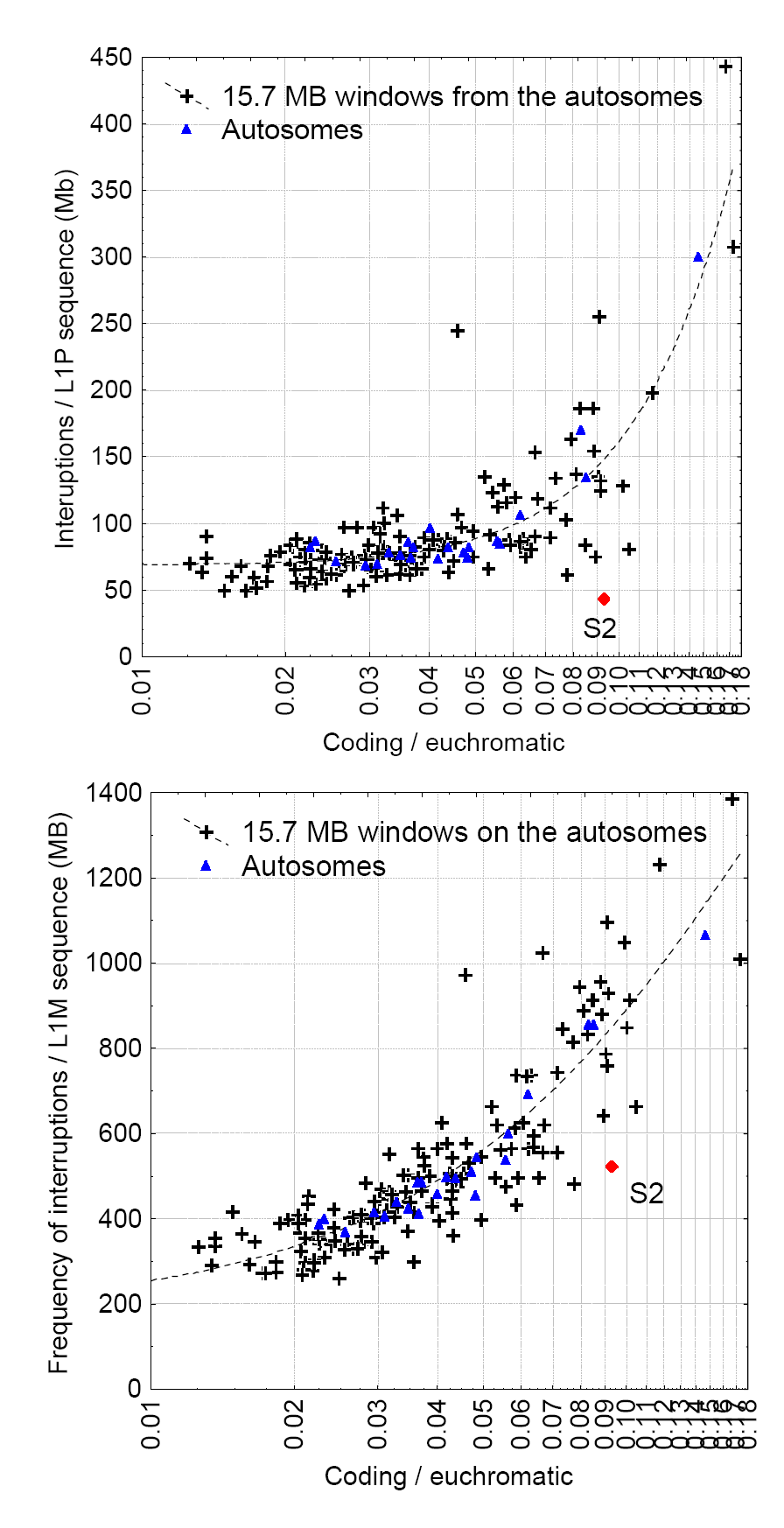

Supplement: Figure S3 — Regressions between the frequency of interruptions and fraction of coding sequence for 15.7 MB regions in the genome and the S2. (0.21 MB TIF) [file pgen.1000172.s003.tif]
